# Pharmaceutical Payments to Authors of Dermatology Guidelines After Publication

**DOI:** 10.2196/37749

**Published:** 2022-06-20

**Authors:** Torunn E Sivesind, Mindy D Szeto, Jarett Anderson, Jalal Maghfour, Maya Matheny, Quan Nguyen Minh Le, Michael Kamara, Robert Dellavalle

**Affiliations:** 1 Department of Dermatology University of Colorado Anschutz Medical Campus Aurora, CO United States; 2 Arizona College of Osteopathic Medicine Glendale, AZ United States; 3 Department of Dermatology Henry Ford Hospital Detroit, MI United States; 4 Santa Clara University Santa Clara, CA United States; 5 University of Missouri School of Medicine Columbia, MO United States; 6 Dermatology Service Rocky Mountain Regional Veterans Affairs Medical Center Aurora, CO United States

**Keywords:** practice guidelines, conflict of interest, industry, dermatology, pharmaceutical, financial disclosures, guideline development, disclosure, transparency, bias, dermatologic drugs, dermatology guidelines, financial interest, payments, industry payments

Clinical practice guidelines (CPGs) play increasingly vital and influential roles in clinical decision-making, optimization of patient care, and establishment and assessment care quality standards, and can affect insurance coverage. Oftentimes, CPG author expertise is sought by insurance and pharmaceutical companies, creating industry-physician relationships that may influence physicians’ professional decisions. This is known as a conflict of interest (COI). Previous studies [1,2] provide strategies for reducing COI impact on guideline development (eg, restricting voting on final recommendations by committee members with COIs [1], requiring conflict-free periods prior to participation in guideline development [2]). In a June 2020 statement, the American Academy of Dermatology (AAD) announced revisions to its guideline development process, specifying that at least 51% of those authoring guidelines be nonconflicted (ie, no relevant financial COIs) and requiring nonconflicted authors to remain so for the entire guideline development process (ie, no new relevant industry relationships initiated during development) [3]. CPG development ends when the draft guideline is approved by the AAD’s Board of Directors and submitted for publication [4]. The AAD requires disclosure of financial interests occurring within the 2-year period prior to CPG authorship [5]. Although a prior study[6] demonstrated that former Food and Drug Administration committee members frequently received payments from the industry after the approval of dermatologic drugs, to our knowledge, there exists no similar exploration of industry payments to authors of recently published AAD guidelines.

Post hoc general industry payments to AAD guideline authors in the period shortly following guideline publication (defined as publication year and 1 subsequent year) were analyzed. We reviewed all current AAD CPGs, including acne vulgaris, atopic dermatitis, keratinocyte carcinoma (basal cell carcinoma and squamous cell carcinoma, same guideline authors), melanoma, psoriasis, and surgery, with publication dates spanning from 2013 to 2018. General payments made by companies to each CPG author were extracted and aggregated from publicly available data in the Centers for Medicare and Medicaid Services Open Payments database [7]. The psoriasis guideline was excluded from further analysis because, unlike the other guidelines, it was published after the recent changes to the AAD’s COI policy for guideline authors, and Open Payments data was only available through 2020. The Food and Drug Administration Orange [8] and Purple [9] Book databases were searched to identify companies (and subsidiaries, according to US Securities and Exchange Commission filings) that were manufacturers of CPG drugs.

Of the 6 dermatology CPGs (Table 1), total payments to CPG authors by pharmaceutical companies manufacturing CPG-recommended drugs ranged from $46,554 (melanoma) to $1,374,780 (acne).

Of 99 unique CPG authors, 56 (57%) received at least one payment from a company responsible for a CPG-recommended drug (range 39%-74% across guidelines) (Table 2). A total of 22 (22%) received ≥$10,000 and 10 (10%) received ≥$50,000.

Overall, AAD CPG authors received substantial industry payments from companies with financial interests in the guideline recommendations, corroborating previous studies [10]. Industry payments occurring in the early postpublication period were received by more than 51% of the authors of CPGs on atopic dermatitis, acne, and surgery. Efforts to improve the transparency of author disclosures and minimize commercial bias are encouraged, and future studies should assess the impact of the recently implemented changes to the AAD’s guideline development.

**Table 1:** Pharmaceutical payments^a^ to American Academy of Dermatology (AAD) clinical practice guideline (CPG) authors.

Rank	Atopic dermatitis guideline drug company	Payments to authors ($)	Acne vulgaris guideline drug company	Payments to authors ($)	Local anesthesia for dermatologic surgery guideline drug company	Payments to authors ($)	BCC^b^ and SCC^c^ guideline drug company	Payments to authors ($)	Melanoma guideline drug company	Payments to authors ($)
1	Medimetriks Pharmaceuticals	223,630.70	Galderma (and subsidiaries)	453,849.76	Teva (and subsidiaries)	53,630.20	Lilly (and subsidiaries)	123,696.59	Merck	21,546.61
2	Pfizer (and subsidiaries)	170,815.50	Abbvie (and subsidiaries)	429,684.33	Purdue Pharma	12,837.50	Novartis (and subsidiaries)	73,842.97	Bristol Myers Squibb (and subsidiaries)	14,548.21
3	Novartis (and subsidiaries)	147,242.97	Valeant Pharmaceuticals	209,588.28	Sun Pharma (and subsidiaries)	9178.76	Pfizer (and subsidiaries)	16,490.66	Novartis	7271.78
4	Celgene Corporation	76,003.29	Bayer	110,725.18	Genentech	8394.02	Pierre Fabre Pharmaceuticals	6842.92	Bausch (and subsidiaries)	2238.43
5	Valeant Pharmaceuticals	47,953.71	Pfizer (and subsidiaries)	85,442.24	Novartis (and subsidiaries)	5495.04	Bausch (and subsidiaries)	4496.13	Roche (and subsidiaries)	949.61
6	Galderma (and subsidiaries)	35,197.51	Sanofi (and subsidiaries)	27,533.66	Pfizer (and subsidiaries)	2561.25	Biofrontera	540.67	—^d^	—
7	Lilly (and subsidiaries)	29,300.02	Dr Reddy's Laboratories (and subsidiaries)	19,922.14	Bristol Myers Squibb (and subsidiaries)	1185.94	Smith & Nephew (and subsidiaries)	325.56	—	—
8	Abbvie (and subsidiaries)	27,875.65	Novartis (and subsidiaries)	19,880.38	Abbott Laboratories	325.00	Sun Pharma (and subsidiaries)	231.67	—	—
9	Sanofi-Aventis	24,989.05	Almirall (and subsidiaries)	5565.86	Bayer	117.13	Taro Pharmaceuticals	108.78	—	—
10	Merck (and subsidiaries)	22,693.38	Sun Pharma (and subsidiaries)	4235.73	Valeant Pharmaceuticals	73.87	Genentech	61.92	—	—
11	Astellas Pharma	18,777.96	Janssen (and subsidiaries)	2783.54	Merck	45.71	Almirall	33.51	—	—
12	Roche (and subsidiaries)	17,618.10	Taro	2472.86	Lilly	35.58	Amgen	14.33	—	—
13	Ranbaxy	16,385.19	Exeltis	1750.00	Promius Pharma	19.14	—	—	—	—
14	GlaxoSmithKline	16,102.24	Lilly	650.40	—	—	—	—	—	—
15	Aqua Pharmaceuticals	13,812.70	Merz (and subsidiaries)	565.31	—	—	—	—	—	—
16	Taro Pharmaceuticals	10,010.42	Lupin Pharmaceuticals	49.66	—	—	—	—	—	—
17	Bayer	9000.00	Biofrontera	30.99	—	—	—	—	—	—
18	Dr Reddy's Laboratories (and subsidiaries)	6680.09	Shire	22.15	—	—	—	—	—	—
19	Leo Pharma (and subsidiaries)	5618.60	Teva	14.86	—	—	—	—	—	—
20	Medimmune	1844.42	Arbor	12.77	—	—	—	—	—	—
21	Teva (and subsidiaries)	1394.09	—	—	—	—	—	—	—	—
22	UCB SA	358.00	—	—	—	—	—	—	—	—
Total payments to authors		923,303.59	—	1,374,780.10	—	93,899.14	—	226,685.71	—	46,554.64

^a^General payment data from Open Payments was totaled for each AAD CPG author in the year of CPG publication and the subsequent year. General payments include payments or other transfers of value that were not made in connection with a research agreement or research protocol. Company subsidiaries were determined according to recent US Securities and Exchange Commission filings.

^b^BCC: basal cell carcinoma.

^c^SCC: squamous cell carcinoma.

^d^N/A: not applicable.

**Table 2:** American Academy of Dermatology (AAD) clinical practice guideline (CPG) authors receiving payments^a^ from pharmaceutical companies manufacturing CPG-recommended drugs.

	Atopic dermatitis	Acne vulgaris	Local anesthesia for dermatologic surgery	BCC^b^ and SCC^c^	Melanoma	Total unique guideline authors
Total payments to guideline authors ($)	923,303.59	1,374,780.10	93,899.14	226,685.71	46,554.64	2,665,223.18
Total guideline authors, n	23	22	14	31	16	99
Authors receiving payments, n (%)	17 (74)	14 (64)	8 (57)	12 (39)	7 (44)	56 (57)
Mean payment to authors receiving payments ($)	54,311.98	98,198.58	11,737.39	18,890.48	6650.66	47,593.27
Median payment to authors receiving payments ($)	9319.02	23,475.75	1940.41	256.34	2194.87	4939.40
Authors receiving payments ≥$10,000, n (%)	7 (30)	11 (50)	1 (7)	3 (10)	1 (6)	22 (22)
Authors receiving payments ≥$50,000, n (%)	3 (13)	5 (23)	1 (7)	1 (3)	0 (0)	10 (10)

^a^General payment data from Open Payments was totaled for each AAD CPG author in the year of CPG publication and the subsequent year. General payments include payments or other transfers of value that were not made in connection with a research agreement or research protocol.

^b^BCC: basal cell carcinoma.

^c^SCC: squamous cell carcinoma.

## Abbreviations

AAD: American Academy of Dermatology
COI: conflict of interest
CPG: clinical practice guideline
JAAD: *Journal of the American Academy of Dermatology*
JID: *Journal of Investigative Dermatology*
